# Discovery of Herbal Pairs Containing *Gastrodia elata* Based on Data Mining and the Delphi Expert Questionnaire and Their Potential Effects on Stroke through Network Pharmacology

**DOI:** 10.1155/2020/4263591

**Published:** 2020-04-05

**Authors:** Rongrong Zhou, Yan Zhu, Wei Yang, Fengrong Zhang, Junwen Wang, Runhong Yan, Shihuan Tang, Zhiyong Li

**Affiliations:** ^1^College of Pharmacy, Minzu University of China, Beijing 100081, China; ^2^Shanxi University of Chinese Medicine, Taiyuan 030619, China; ^3^Institute of Information on Traditional Chinese Medicine, China Academy of Chinese Medical Sciences, Beijing 100700, China; ^4^Institute of Basic Research in Clinical Medicine, China Academy of Chinese Medical Sciences, Beijing 100700, China; ^5^Shandong University of Traditional Chinese Medicine, Jinan 250355, China; ^6^Institute of Basic Theory of Chinese Medicine, Chinese Academy of Chinese Medicine Sciences, Beijing 100700, China; ^7^Institute of Chinese Materia Medica, China Academy of Chinese Medical Sciences, Beijing 10070, China

## Abstract

**Background:**

Traditional Chinese medicine (TCM) formulae can be regarded as a source of new antistroke drugs. The aim of this study was to discover herbal pairs containing *Gastrodia elata* (Tianma, TM) from formulae based on data mining and the Delphi expert questionnaire. The proposed approach for discovering new herbal combinations, which included data mining, a clinical investigation, and a network pharmacology analysis, was evaluated in this study.

**Methods:**

A database of formulae containing TM was established. All possible herbal pairs were acquired by data mining association rules, and herbal pairs containing TM were screened according to the *Support* and *Confidence* levels. Taking stroke as the research object, the relationships between herbal pairs containing TM and stroke were explored by the Delphi expert questionnaire and statistical methods. To explore the effects of herbal pairs containing TM on stroke, a network pharmacology analysis was performed to predict core targets, biological functions, pathways, and mechanisms of action.

**Results:**

A total of 1903 formulae containing TM, involving 896 Chinese herbal medicines (CHMs) and 126 herbal pairs containing RG, were analyzed by association rules. A total of 27 herbal pairs were further screened according to the *Support* and *Confidence* levels. Twelve herbal pairs containing RG were added according to the expert questionnaires. Weightiness analysis showed that 9 groups of core herbal pairs contained RG, including TM-QX, TM-JH, TM-CX, TM-GG, TM-SJM, TM-JC, TM-SCP, TM-MJZ, and TM-GT. Two core herbal pairs, TM-JH and TM-CX, were randomly screened to explore their network pharmacological mechanisms in stroke. The important biological targets for network pharmacological analysis of TM-CX and TM-JH related to stroke were PTGS2, ACE, APP, NOS1, and NOS2. An herbal pair-compound-core target-pathway network (H-C-T-P network) was established, and arginine biosynthesis, arginine and proline metabolism, and the relaxin signaling pathway were identified by enrichment analysis.

**Conclusion:**

The herbal pairs of TM-CX and TM-JH obtained from data mining and the expert investigation were found to have effects of preventing and treating stroke through network pharmacology. This could be a viable approach to uncover hidden knowledge about TCM formulae and to discover herbal combinations with clinical and medicinal value based on data mining and questionnaires.

## 1. Introduction

Traditional Chinese medicine (TCM) in China has led to the accumulation of expansive theoretical knowledge and clinical experiences over the past thousands of years. TCM formulae are formed from herbal medicines, animal medicines, minerals, and other traditional medicines to enhance curative effects and reduce adverse reactions. The book named *Prescriptions for Fifty-two Diseases* (52 Bingfang, 206 BC∼8 AD) is one of the oldest existing works recording medical formulae in China. Since then, *Yellow Emperor's Inner Canon* (Huangdi Neijing, 770 BC∼220 AD) laid the foundation for the formula theories of TCM, and *Treatise on Cold Pathogenic and Miscellaneous Diseases* (Shanghan Zabing Lun, 200 AD∼210 AD) is another representative work [1] containing approximately 100,000 formulae recorded in various studies. The numerous formulae are of great value in clinical research of TCM [1]. Herbal pairs are the most basic and simplest element in formulae that represent the concentrated expression of herbal compatibility. *Shennong's Classic of Materia Medica* (Shennong Bencao Jing, 100 BC∼46 BC) provided an important theoretical basis for herbal compatibility [2]. The principle of “monarch, minister, assistant, and guide” described in *Yellow Emperor's Inner Canon* has been applied to herbal compatibility and provides guidance for clinical practice [3].

The compatibility of Chinese herbal medicines (CHMs) can enhance the efficacies of herbs and reduce their possible toxicities [4, 5]. The reasonable compatibility of CHMs and the compatibility law of formulae have become a foundation in the exploration of TCM [6, 7]. GASTRODIAE RHIZOMA (*Gastrodia elata* Bl., Tianma, TM) is a main herb that can prevent and treat stroke [8], a destructive neurological condition that can lead to death and long-term disability. Stroke has brought about a huge burden to society [9]. Increasing experimental and evidence-based medical evidence shows that TM compatible with other CHMs can be used to treat and prevent stroke and repeat stroke. TM and *Uncaria rhynchophylla* (Gouteng, GT) have been confirmed to modulate the antioxidant system and antiapoptotic genes in oxygen glucose-deprived neuronal differentiated PC12 cells and in middle cerebral artery occlusion (MCAO) rats [10], benefiting the treatment of stroke or repeat stroke. Therefore, for the development of new drugs, it is of great significance to identify more herbal pairs containing TM from formulae or clinical prescriptions that can effectively prevent ischemic stroke and to explain their mechanism of antistroke.

The application of data mining, bioinformatics, network pharmacology, and other emerging technologies has greatly changed the scientific understanding of TCM and has played major roles in addressing the complexity of TCM [11–13]. Data mining is considered an important tool for discovering the potential associations of formulae [14]. TCM herbal formulae have been considered multicomponent and multitarget therapeutics, which can potentially meet the demand to treat a number of complex diseases, and network pharmacology methods can be used to gain a priori knowledge about the combination rules embedded in formulae [15]. Thus, these are considered emerging and powerful approaches for revealing the underlying complex interactions between formula and cellular proteins [16]. Studies have suggested employing data mining and TCM network pharmacology approaches as a new research paradigm for translating TCM from an experience-based medicine system to an evidence-based medicine system, which would accelerate drug discovery from TCM [17].

In this study, all herbal pairs containing TM were obtained from formulae by data mining, and herbal pairs that could effectively prevent and treat stroke were further identified through a clinical expert questionnaire. Then, the potential effects of the herbal pairs containing TM on stroke were predicted and evaluated by analyzing their roles in the stroke target network by network pharmacology. This study provides new research ideas and strategies for discovering new Chinese herbal pairs and potential herbal combinations.

## 2. Materials and Methods

### 2.1. Data Sources

The data used in this study were obtained from the Chinese Traditional Medicine Database, which contains 84464 formulae from more than 710 ancient and modern documents and was built by the Information Institute of China Academy of Chinese Medical Sciences (http://cintmed.cintcm.com/cintmed/main.html).

### 2.2. Inclusion Criteria

The formulae underwent data cleaning and transformation to be suitable for data mining [18]. Formulae and CHMs were required to meet the following criteria: (1) the formula composition contains TM; (2) the formula originated from ancient books of TCM before 1911 AD with a clear source; (3) the administration route of the formula was oral; (4) the formula has a clear composition of herbs (here, “herbs” refer not only to plants but also to animal sources and minerals with treatments effects); and (5) the names of CHMs were standardized according to *Chinese Pharmacopoeia* (2015 edition, China Pharmaceutical Science and Technology Publishing House) and *Great Compendium of Chinese Medicines* (second edition of 2014, Shanghai Science and Technology Publishing House), such as changing the herbal name “Wu Shi” to “Niu Bang Zi” (ARCTII FRUCTUS) and changing the name “Dong Chong Cao” to “Dong Chong Xia Cao” (CORDYCEPS).

### 2.3. Data Mining Process and Methods

High-frequency herbal pairs were discovered using the algorithm of association rules for arules package in R language [19]. The algorithm of association rules is the most common method for data mining of TCM formulae; it can be used to investigate CHM compatibility patterns and to reflect the interdependence and relationship between variables [20]. The high-frequency herbal pairs containing TM were screened and retained according to *Support*, given that *Lift* > 1 and *Confidence* ≥ 90% [21]. A higher value of *Support* reflects that the herb is more in line with herbal pair dependence. The higher the *Support* is, the more consistent the relationship of herbs.

*Support* is a measure that reflects how frequently the rule occurs in the database. In this study, *Support* indicates the probability of herb *X* and *Y* existing simultaneously:

(1) $$\begin{array}{c} Support\left(X->Y\right)=P\left(X\cup Y\right). \end{array}$$

*Confidence* refers to the ratio of the probability of the coexistence of herbs *X* and *Y* to the existence of herb *X* in a dataset, reflecting the closeness of the relationship between them:

(2) $$\begin{array}{c} Confidence\left(X->Y\right)=\frac{Support\left(X\cup Y\right)}{Support\left(X\right)}. \end{array}$$

*Lift* refers to the ratio of the probability of containing both *X* and *Y* of CHM to the probability of containing only *Y* without *X*. *Lift* is greater than 1, indicating that the association rules are meaningful.

### 2.4. Delphi Process

The Delphi process is an objective method to mine formula compatibility with data mining. In the mining of herbal pairs for antistroke treatment, attention should be paid to both deciphering the potential objective laws of ancient formulae and the clinical application. The Delphi method is considered an ideal method for reaching consensus and is essentially an anonymous feedback inquiry method. Two rounds of consultation were performed in this study. The questionnaire based on the Delphi method involved five aspects.

#### 2.4.1. Group

The research group was set up to investigate the clinical use of TM in the prevention and treatment of stroke.

#### 2.4.2. Object

Referring to international experience, the questionnaire follows the general principles of integrity, simplicity, guidance, comparability, uniformity, operability, practicality, regionality, authority, and representativeness [22]. Herbal pairs containing TM included the high-frequency herbal pairs obtained by data mining and the clinical herbal pairs obtained from a literature investigation. Clinicians were required to recommend the commonly used herbal pairs containing TM in the first round of the questionnaire, and the herbal pairs were added to the second round. In the whole process of questionnaire consultation, clinicians should evaluate the relationship of herbal pairs and stroke from “herbal pairs correspond to clinical syndrome type of stroke” and “seven features of compatibility” [23].

#### 2.4.3. Questionnaire Design Form

The clinicians evaluated whether herbal pairs containing TM were commonly used in preventing and treating stroke, and the degree of use of herbal pairs was graded according to a Likert scale (Table 1).

#### 2.4.4. Selection Conditions of Clinicians

Conditions for screening clinicians were defined, and the questionnaires were sent to the clinicians who met the conditions and agreed to participate [24] (Table 2). The selection conditions meet the first 4 conditions and any of 5, 6, and 7 conditions. All the completed questionnaires were received within one month, and statistical processing was carried out. The names of the respondents were not revealed throughout the whole process.

#### 2.4.5. Feedback

In the second round of the questionnaire, the judgment results and opinions of the clinicians obtained from the first round were provided back to the clinicians, and they were asked whether they had changed their original judgments, and clinicians in the second round were invited to evaluate the new herbal pairs.

### 2.5. Statistical Analysis

#### 2.5.1. Statistical Analysis of the Delphi Process

The indexes of the Delphi questionnaire were quantitatively analyzed by the descriptive statistics module, the reliability analysis module, and the nonparametric tests module of SPSS 19.0. The maximum, minimum, mean, standard deviation, median, frequency, coefficient of variation, reliability, and Kendall's W harmony coefficient were analyzed [25] (Table 3).

#### 2.5.2. Weightiness Analysis

In this experiment, the data mining association rule and the Delphi expert investigation method were used to obtain the herbal pairs containing TM for stroke. The two groups of data were standardized by the dimensionless method, and the weightiness analysis was performed according to a certain proportion [26].

The dimensionless calculation formula for the data *x*_1_, *x*_2_,…, *x*_*n*_ is as follows:

(3) $$\begin{array}{c} y_{i}=\frac{x_{i}-x_{\min}}{x_{\max}-x_{\min}}\times 100\%. \end{array}$$

The new sequence *y*_1_, *y*_2_,…, *y*_*n*_ ∈ [0, 1] is dimensionless. The herbal pairs in the association rule group (group 1) were subjected to dimensionless treatment according to the *Support*, and the herbal pairs in the Delphi expert investigation group (group 2) were subjected to dimensionless treatment according to the degree of herbal pair usage.

Weightiness analysis calculation formula is given by

(4) $$\begin{array}{c} W_{i}=\frac{y_{iA}+y_{iB}}{2}\times 100, \end{array}$$

where *y*_*iA*_ refers to the dimensionless value of herbal pairs (i) obtained by the data mining method (Group A), and *y*_*iB*_ refers to the dimensionless value of herbal pairs (i) obtained by the Delphi expert survey method (Group B). This study considered data mining and expert questionnaires to be equally important. The index function is used to randomly select two groups of herbal pairs for network pharmacology research.

### 2.6. Network Pharmacological Analysis of Herbal Pairs Containing RG

The molecular mechanism of herbal pairs containing TM was predicted and confirmed by network pharmacology. To facilitate this research, herbal pairs with high midweight analysis values were screened, and the mechanism of action of the herbal pairs on stroke was analyzed by using the network pharmacology method.

#### 2.6.1. Compound Preparation

Compounds of herbal pairs were collected from the Traditional Chinese Medicine Systems Pharmacology Database (TcmSP™, http://lsp.nwu.edu.cn), the Traditional Chinese Medicine Database@Taiwan (TCM Database@Taiwan, http://tcm.cmu.edu.tw/), the Traditional Chinese Medicine Integrated Database (TCMID, http://www.megabionet.org/tcmid/), and The Encyclopedia of Traditional Chinese Medicine (ETCM, http://www.ehbio.com/ETCM/) [27], and the compounds of CHM were supplemented and validated by the PubChem Compound database.

#### 2.6.2. Screening of Potential Active Compounds of Herbs

DruLiTO is a tool for calculating the druglikeness of compounds from herbal pairs and known drugs, which follows Lipinski's “rule of five” (i.e., a molecule with a molecular mass less than 500 Da, no more than 5 hydrogen bond donors, no more than 10 hydrogen bond acceptors, no more than 10 rotatable bonds, and an octanol-water partition coefficient log *P* not greater than 5) [28]. SwissADME (http://www.swissadme.ch/) was used to screen the components of herbal pairs that are easily absorbed by the intestine (GI) and that permeate by the blood-brain barrier (BBB). The properties of the components were demonstrated by BOILED-Egg, a skilled method in SwissADME [29].

#### 2.6.3. Target Prediction for Potential Active Molecule Compounds

The SwissTargetPrediction platform [30] was used to predict the candidate targets of active molecule compounds in herbs, and *Homo sapiens* was chosen by default.

#### 2.6.4. Building the Disease Target Database

Taking “apoplexy,” “apoplexia,” “stroke,” and “ischemic stroke” as themes, the disease targets were searched in OMIM (Online Mendelian Inheritance in Man, http://omim.org/), Human Phenotype Ontology (HPO, http://human-phenotype-ontology.github.io/), and TCMIP (Internet-based Computation Platform for IP of TCM, http://www.tcmip.cn). The UniProt database was used to normalize the targets and convert them into *Homo sapiens* gene names.

#### 2.6.5. Cluster Analysis of Core Proteins

Two groups of candidate targets of herbal pairs containing RG were selected to interact with the targets of stroke. The common targets were then processed by String (https://string-db.org/) to obtain the protein-protein interaction (PPI). The core targets were obtained by the degree calculated according to Network Analyzer of Cytoscape 3.2.1 and were clustered by the MCODE module. The parameter K-core, which determined the size of the recognition module, was set to 2 in the MCODE module (the identified module contains at least three edges).

#### 2.6.6. Biological Function and Enrichment Pathway Analysis of Identifiable Targets

The Gene Ontology (GO) annotation of core target bubble charts was mapped using an Omicshare cloud platform (https://www.omicshare.com/tools/Home/Report/goenrich). The list of genes was submitted, the species “*Homo sapiens*” was selected for GO annotation enrichment, and the functions were screened with a cutoff *P* < 0.001.

#### 2.6.7. Construction of Herbal Pair-Compound-Core Target-Pathway Networks

The “herbal pair-compound-core target-pathway” network (H-C-T-P network) was constructed with Cytoscape 3.2.1. Starting from the analysis of core targets, the related active small molecule compounds of herbal pairs and metabolic pathways of diseases were correlated to form the molecular mechanism action network of herbal pairs [31]. The String database was used to enrich the molecular pathways from all core targets of herbal pairs for treating stroke, and these pathways were screened with a cutoff FDR (false discovery rate) < 0.05.

## 3. Results

### 3.1. Association Rule Analysis Results

A total of 896 herbs were screened from 1903 formulae containing TM, and their occurrence frequency was 15918 times. SAPOSHNIKOVIAE RADIX (*Saposhnikovia divaricata* (Turcz. Schischk, Fangfeng, FF), SCORPIO (*Buthus martensii* Karsch Quanxie, QX), MOSCHUS (*Moschus berezovskii* Flerov, Shexiang, SX), TYPHONII RHIZOMA (*Typhonium giganteum* Engl, Baifuzi, BFZ), and other CHMs were found to have high frequencies in our study (Table 4).

## 4. Investigation Results of Herbal Pairs Containing TM Based on the Delphi Expert Questionnaire

### 4.1. Expert Basic Survey

According to the expert screening criteria, the respondents of the returned questionnaires were evaluated based on their academic qualifications, professional titles, and working years. The questionnaires from respondents who did not meet the criteria for expert selection were not included in the statistical category (Table 5).

### 4.2. Expert Positive Coefficient

In the first round, 24 questionnaires were distributed in May 2017, and 18 valid questionnaires were received back (the positive coefficient was 75%). In the second round, 21 questionnaires were distributed from July to September 2017, and 20 valid questionnaires were collected (the positive coefficient was 94.73%). Both rounds of questionnaires were effective consultations.

### 4.3. Distribution of Expert Opinions

The usage of TM and its herbal pairs by clinicians was the best evidence reflecting the application value of TM (Table 6).

### 4.4. Degree of Coordination of Expert Opinions

The Kendall coefficient was 0.571 (*χ*^2^ = 267.06; *P* < 0.01) in the first round of the expert questionnaire and 0.414 (*χ*^2^ ^* *^=  89.395; *P* < 0.01) in the second round of the expert questionnaire. The Kendall coefficients of the two rounds of the expert questionnaire showed that the clinicians did not have a high overall coordination in their responses regarding the clinical application of TM.

The coefficient of variation of 13 herbal pairs containing TM was less than 15% in the two rounds of the questionnaire (Table 7). TM ⇒ DZ (10.55%), TM ⇒ GT (11.2%), TM ⇒ JC (11.32%), TM ⇒ DL (12.12%), TM ⇒ BS (12.99%), TM ⇒ CX (14.18%), TM ⇒ QH (14.38%), and TM ⇒ QX (14.99%) were less than 15% in the first round of the questionnaire. TM ⇒ JH (9.76%), TM ⇒ GG (10.88%), TM ⇒ JMZ (13.93%), TM ⇒ SCP (13.85%), and TM ⇒ SJM (13.51%) were less than 15% in the second round of the questionnaire.

### 4.5. Reliability of the Expert Questionnaire

The reliability of the expert consultation questionnaire was calculated by the reliability analysis in the “measurement” module of SPSS 21.0. Cronbach's alpha coefficients of the two rounds of questionnaires were 0.900 and 0.813, respectively, which showed that the indexes were reliable in the two rounds.

### 4.6. Weightiness Analysis Results

In this study, TM-QX, TM-FF, TM-JC, TM-CX, TM-QH, TM-BFZ, and TM-JH had higher weight values (Table 8).

## 5. Network Pharmacological Analysis

According to the above results, TM ⇒ QX, TM ⇒ FF, TM ⇒ JC, TM ⇒ CX, TM ⇒ QH, TM ⇒ BFZ, and TM ⇒ JH had relatively high weight values. To facilitate network pharmacological analysis, two pairs of herbal pairs were selected by the index function for the follow-up study. TM-CX and TM-JH were randomly screened out.

### 5.1. Potential Active Molecule Compounds of Herbal Pairs Containing TM

**Figure 1:**
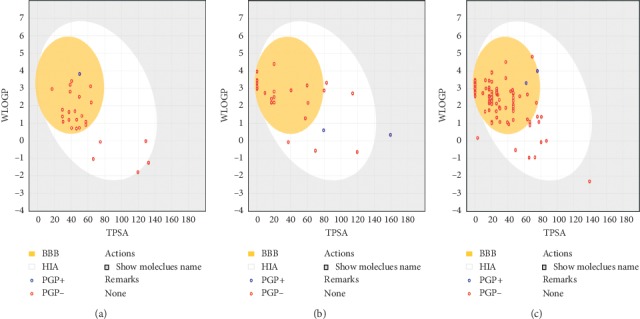
BOILED-Egg chart of CHM. (a) TM. (b) JH. (c) CX. Note: the white area shows the physicochemical space of the molecule with the highest probability of absorption in the gastrointestinal tract, while the yellow area (yolk) shows the physicochemical space of the molecule with the highest probability of penetration into the brain. Yellow and white areas are not mutually exclusive. Blue dots represent molecules that are better absorbed by the intestine; red dots represent molecules that are more permeable to the brain.

Potential active molecule compounds were screened by SwissADME online tools (Figure 1), including 115 from CX, 28 from JH, and 24 from TM. Prediction by the BOILED-Egg chart showed that TM, CX, and JH had good gastrointestinal absorption (blue) or blood-brain permeation (red), which are more inclined to permeate the BBB.

### 5.2. Target Prediction and Analysis

**Figure 2:**
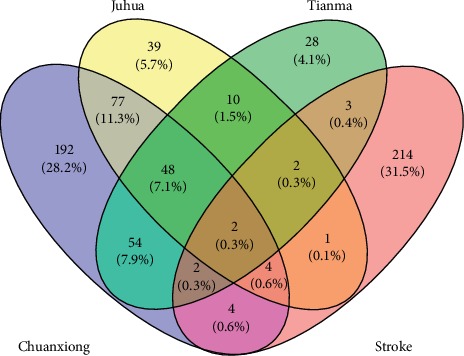
Venny diagram of both the targets of stroke and herbal pairs.

A total of 680 targets of active small molecule compounds of TM, CX, and JH were predicted based on the principle of chemical structure similarity, including 383 targets related to CX, 184 targets related to JH, and 149 targets related to TM. A total of 232 stroke-related targets were screened by the OMIM and HPO disease databases. A Venny chart was used to show the common predictive targets and stroke targets (Figure 2). Among the common targets with herbal pairs, there were 106 common targets related to TM-CX and 62 common targets interrelated with TM-JH. Among the common stroke targets, there were 12 common targets relevant to CX, 9 common targets relevant to TM, and 9 common targets relevant to JH.

### 5.3. Common Target Clustering Analysis

**Figure 3:**
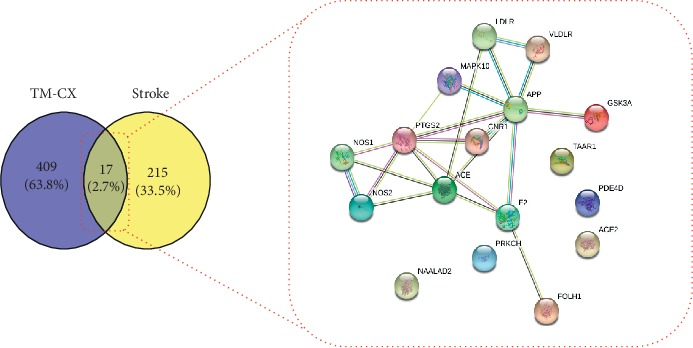
After constructing the interaction map between the known targets of stroke and the potential targets of TM-CX, a protein-protein interaction (PPI) network was developed to identify the 17 interactive core targets.

**Figure 4:**
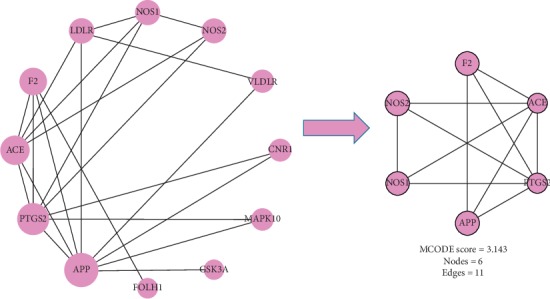
Cluster analysis from the MCODE plugin of Cytoscape resulted in PPI networks.

**Figure 5:**
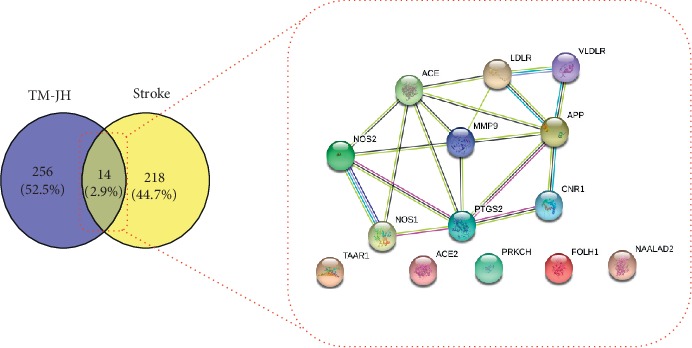
After constructing the interaction map between the known targets of stroke and the potential targets of TM-JH, the protein-protein interaction (PPI) network was produced to identify the 14 interactive core targets.

**Figure 6:**
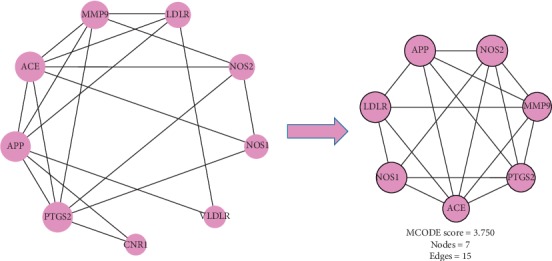
Cluster analysis from the MCODE plugin of Cytoscape resulted in PPI networks.

The results of cluster analysis showed that there were 17 common targets between potential targets of TM-CX and known targets of stroke (Figure 3), of which 6 were core targets by cluster analysis (Figure 4), 14 were common potential targets of TM-JH and known targets of stroke (Figure 5), and 7 were core targets by cluster analysis (Figure 6). The targets such as PTGS2, ACE, APP, NOS1, and NOS2 were all in the datasets of CX and JH (Tables 9∼10).

### 5.4. Biological Function Enrichment Analysis of Core Targets

**Figure 7:**
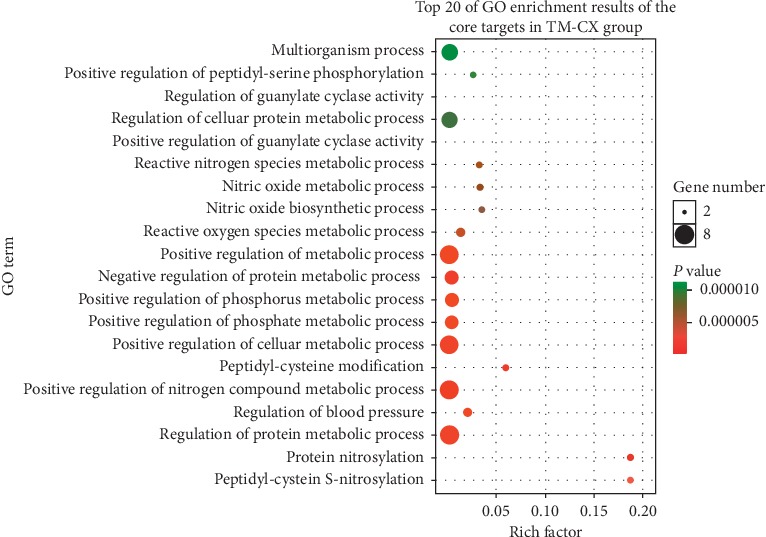
Biological functions and molecular pathways from all core targets relevant to TM-CX in stroke, with the top 20 biological processes screened and classified.

We obtained 1890 GO annotations about the core targets relevant to TM-CX, including the regulation of protein metabolic processes, positive regulation of nitrogen compound metabolic processes, negative regulation of protein metabolic processes, positive regulation of cellular metabolic processes, positive regulation of metabolic processes, and multiorganism processes. A total of 2085 GO annotations were obtained about core targets relevant to TM-JH, including the regulation of protein metabolic processes, positive regulation of nitrogen compound metabolic processes, negative regulation of metabolic processes, positive regulation of metabolic processes, positive regulation of phosphorus metabolic processes, and positive regulation of phosphate metabolic processes. The top 20 GO enrichment results are shown (Figures 7∼8).

### 5.5. Herbal Pair-Compound-Core Target-Pathway Network

**Figure 9:**
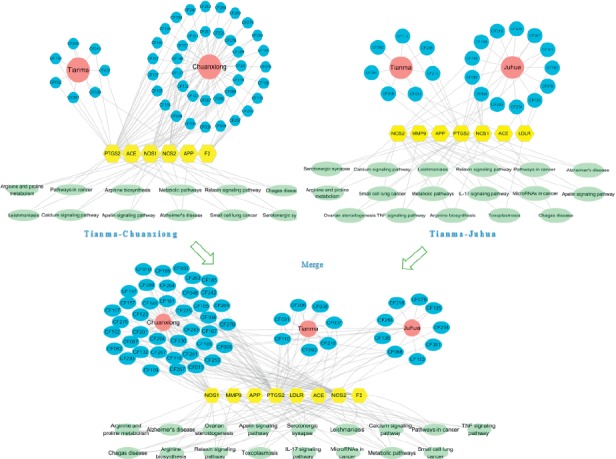
The herbal pair-compound-core target-pathway network.

The pathways corresponding to the core targets relevant to each herbal pair were obtained by the String database, and the “herbal pair-compound-core target-pathway network” (H-C-T-P network) was established by Cytoscape 3.7.1. In the H-C-T-P network (Figure 9), the degree of PTGS2 was the highest, and many compounds in TM, CX, and JH were related to PTGS2. NOS1 and NOS2 were common targets related to CX and JH. APP was a common target related to TM and CX. ACE was a common target related to TM and JH. MMP9 and LDLR were targets related to JH, and F2 was a target related to CX. In the pathway enrichment analysis, important pathways were involved, such as metabolic pathways, cancer pathways, and the relaxin signaling pathway.

## 6. Discussion

TCM is a popular complementary or alternative medicine in Europe, America, and other countries [32, 33] and involves a mature theory of methodology, prescription, and CHMs. Data mining has been successfully used to study the rules of CHMs combined with syndrome differentiation of TCM [13]. TM was first recorded in *Shennong's Classic of Materia Medica* and was listed as a top grade medicine. It is mainly used to prevent and treat headache, dizziness, stroke, migraine, epilepsy, convulsions, neurological headaches, Alzheimer's disease, and other diseases [34]. Studies on compounds and pharmacological effects have shown that TM includes phenols, polysaccharides, sterols, organic acids, and other chemical components. TM is used for a number of effects, including sedative, hypnotic, antiepileptic, anticonvulsant, antianxiety, antidepressive, neural protection, antivertigo, regulation of the circulatory system, anti-inflammatory, analgesic, antioxidative, memory improving, antiaging, antiviral, and antitumoral effects.

In this study, 126 herbal pairs containing TM were obtained based on data mining. A total of 27 herbal pairs were selected for inclusion in the expert questionnaire consultation. Twelve herbal pairs containing TM were supplemented in the process of expert questionnaire survey. Through the weight analysis of the data mining and Delphi expert survey results, 9 core herbal pairs with high weight values that might be more effective for the prevention and treatment of stroke were obtained, namely, TM-QX, TM-JH, TM-CX, TM-GG, TM-SJM, TM-JC, TM-SCP, TM-MJZ, and TM-GT. To verify the reliability of the method used in this research, the herbal pairs TM-CX and TM-JH were selected for evaluation by network pharmacological methods to explore their molecular mechanisms on stroke. The findings suggested that the herbal pairs containing TM might have specific therapeutic effects against stroke. PTGS2, NOS2, NOS1, APP, F2, and ACE were identified as core targets of TM-CX, and APP, ACE, PTGS2, MMP9, LDLR, NOS2, and NOS1 were identified as core targets of TM-JH. Among them, NOS2, NOS1, APP, ACE, and PTGS2 exist in both herbal pairs. Among the active small molecule compounds associated with the core targets, 7 are from TM, 38 are from CX, and 13 are from JH. For example, L-pyroglutamic acid, 3-hydroxybenzoic acid, 4-formyl-2-methoxyphenyl acetate, vanillyl alcohol, p-hydroxybenzyl ethyl ether, bis(4-hydroxybenzyl)ether, and 4-ethoxymethylphenyl-4′-hydroxybenzylether are considered the main active small molecule compounds of TM. Research shows that TM and GT extract can treat hypertension and cerebrovascular disease, obviously improve neurological function, and reduce cerebral infarction [35]. The pathways of arginine biosynthesis, arginine and proline metabolism, and the relaxin signaling pathway have very low FDRs.

According to the *FDR*, three important pathways of TM-CX and TM-JH were predicted in the treatment of stroke: arginine biosynthesis, arginine and proline metabolism, and the relaxin signaling pathway. NOS1 and NOS2 belong to the family of nitric oxide synthases, which are reactive free radicals and act as biologic mediators in several processes in the brain and peripheral nervous system. NOS1 is the main source of NO in the central nervous system and promotes L-arginine catalysis [36, 37]. NOS2 is involved in the inflammatory cascade reaction process after ischemia. The inhibition of NOS2 expression in leukocytes and brain endothelial cells after cerebral ischemia can prolong treatment time and induce long-term neuroprotection [38]. APP is upregulated after acute stroke, chronic cerebrovascular disease, hypoxia, and ischemia brain injury, and it may exert this function by regulating neuronal calcium homeostasis and cell survival [39]. ACE is a potent vasopressor and aldosterone-stimulating peptide that controls blood pressure and vascular homeostasis. ACE plays an important role in the development of cerebrovascular and cardiovascular diseases [40]. PTGS2 is the key enzyme in prostaglandin biosynthesis and plays an important role in modulating motility, proliferation, and resistance to apoptosis. Upregulation of PTGS2 is associated with increased cell adhesion, resistance to apoptosis, and tumor angiogenesis [41].

## 7. Technical Route and Results Diagram

**Figure 10:**
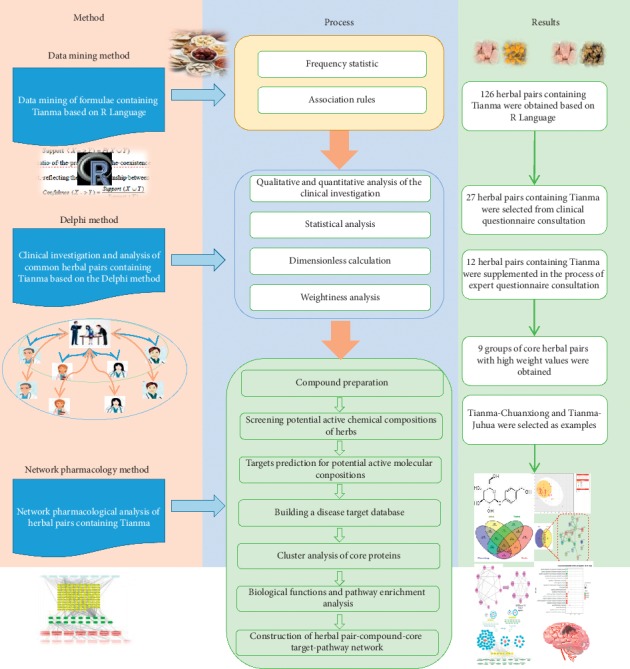
Technical route and results diagram.

The aim of this study was to find a reasonable and effective method to explore herbs that could be compatible with TM for preventing and treating stroke. This study provides a method and technical *Support* for basic research and the clinical application of rational compatibility of TCM for disease prevention and treatment (Figure 10).

## 8. Conclusion

In this study, data mining, an expert investigation, network pharmacology analysis, statistics, and other methods were used to analyze TCM prescriptions in a step-by-step manner. From single variable to multivariate, macroscopic to microscopic, and local to integrated, we provide a comprehensive analysis of TCM prescriptions. We have uncovered hidden knowledge, sought to meet clinical needs, and provided some avenues for the rational integration of TCM, research and development of new herbs or formulae, and clinical prescription.

## 9. Limitations

This study has several potential limitations. First, as we sought to obtain herbal pairs that are compatible with TM as much as possible, it was difficult to uncover deeper tacit knowledge of the compatibility of Chinese medicinal herbs solely by association rules. Second, there are some limitations of the Delphi expert questionnaire: on the one hand, more attention should be paid to the rational design of questionnaires, the principles of expert selection, discussion links with the main issues, statistical methods, and other aspects; on the other hand, modern Chinese medicine lacks a unified basis for judging drug pairs. The screening of herbal pairs containing TM for stroke prevention and treatment depends on clinicians' clinical experiences and subjective knowledge, which makes it difficult for expert consensus to be reached regarding the effectiveness of some infrequently used herbal pairs. Third, the study lacks corresponding outreach work, such as animal and clinical research. Fourth, the experimental samples and scale were small, and the experiment lacked repeated measurements given the limited resources and time. Although subjective errors were avoided as much as possible, some human factors may still exist.

## Figures and Tables

**Figure 8:**
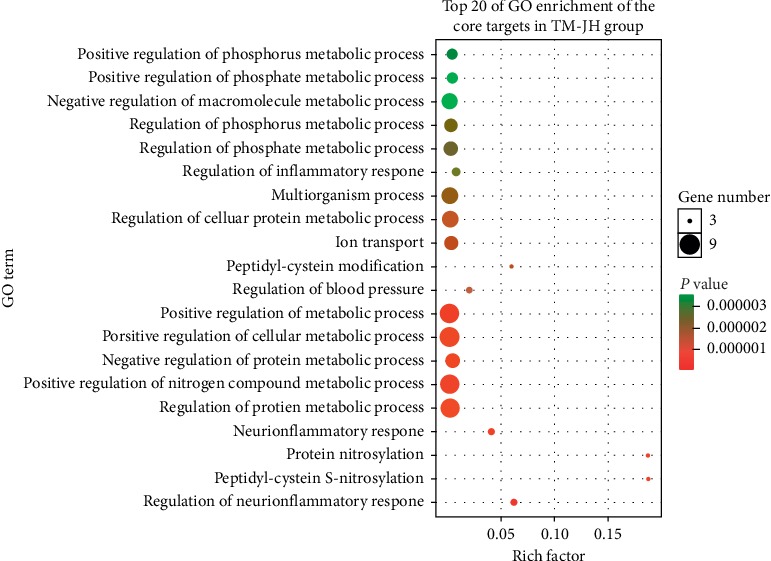
Biological functions and molecular pathways from all core targets relevant to TM-JH in stroke, with the top 20 biological processes screened and classified.

**Table 1:** Likert scale on the clinical application of herbal pairs containing RG.

Degree	Clinical usage level	Grading score (points)
A	Very commonly used	5
B	Commonly used	4
C	Rarely used	3
D	Little or no use	2
E	Unclear	1

**Table 2:** Selection conditions of clinicians.

Selection conditions	Requirements of conditions
1. Hospital level	Grade III Hospital of Traditional Chinese Medicine in China
2. Professional requirements	Internal medicine of TCM
3. Location area	Province with high incidence of stroke in China
4. Working department	Department of Encephalopathy or Neurology of TCM
5. Professional ranks and titles	Deputy Chief Physician and above
6. Educational background	Master of medicine or above
7. Working years	More than 15 years

**Table 3:** Statistical analysis indicators of Delphi method questionnaires.

Statistical indicators	Concept of indicators	Significance of indicators
Positive coefficient (*C*)	Recovery rate of the expert survey and consultation questionnaire (*C* = *n*/*N* ∗ 100%, *n* represents the number of clinicians participating in the questionnaire; *N* represents the total number of clinicians consulted)	A high positive coefficient of the clinicians indicates that the clinicians have a high degree of attention and enthusiasm in participating in this research project

Concentration degree	Reflects the degree of concentration of clinicians' opinions on the relative importance of various indicators; evaluated by the median, mean, and standard deviation and by the median, mean, standard deviation and percentage	The higher the percentage is, the larger the mean, the smaller the standard deviation, and the more important the CHM in the expert evaluation opinions

Degree of coordination (CV)	Reflects the convergence of divergent clinicians' opinions, which is usually expressed by the coefficient of variation and the Kendall harmony coefficient	CV = (*S*/*X*) × 100%; the smaller the coefficient of variation is, the higher the degree of coordination among the clinicians' opinions on herbal pairs containing TM, the smaller the divergence, and the better the convergence
		The Kendall harmony coefficient indicates the overall degree of coordination of clinicians' opinions on herbal pairs. The larger the value is, the higher the degree of coordination of clinicians' opinions (its value ranges from 0 to 1)

Questionnaire reliability (*α*)	Reliability refers to the degree of consistency of the results obtained by repeated measurements of the same object using the same method, which is expressed by Cronbach's alpha	*α* ≥ 0.9 indicates high reliability; 0.8 ≤ *α* < 0.9 indicates acceptable reliability; 0.7 ≤ *α* < 0.8 indicates that some problems may exist; and *α* < 0.7 indicates major problems

**Table 4:** Association rules for herbal pairs containing TM.

Herbal pairs	*Support* (%)	*Confidence* (%)	*Lift*	Count
TM ⇒ FF	44.93	97.38	1.01	855
TM ⇒ QX	39.15	98.68	1.02	745
TM ⇒ SX	32.26	99.19	1.03	614
TM ⇒ BFZ	31.79	99.18	1.03	605
TM ⇒ JC	31.16	98.67	1.02	593
TM ⇒ QH	30.69	97.33	1.01	584
TM ⇒ ZS	28.06	98.16	1.02	534
TM ⇒ TNX	27.59	98.87	1.02	525
TM ⇒ RS	24.65	97.91	1.01	469
TM ⇒ FZ	19.44	98.40	1.02	370
TM ⇒ MH	19.02	99.18	1.03	362
TM ⇒ RG	18.29	98.86	1.02	348
TM ⇒ DH	16.82	97.86	1.01	320
TM ⇒ BX	16.71	97.55	1.01	318
TM ⇒ NH	16.55	98.75	1.02	315
TM ⇒ NX	13.87	97.42	1.01	264
TM ⇒ XX	13.77	97.76	1.01	262
TM ⇒ MX	13.56	97.36	1.01	258
TM ⇒ XH	12.77	98.38	1.02	243
TM ⇒ WSS	12.45	99.58	1.03	237

Notes: only high-frequency herbal pairs containing TM are shown. The names of CHMs are expressed as medical names, Latin names, Pinyin names, and abbreviations. GASTRODIAE RHIZOMA (Rhizoma Gastrodiae, Tianma, TM), SAPOSHNIKOVIAE RADIX (Radix Saposhnikoviae, Fangfeng, FF), SCORPIO (Scorpion, Quanxie, QX), MOSCHUS (Moschus, Shexiang, SX), TYPHONII RHIZOMA (Rhizoma Typhonii, Baifuzi, BFZ), BOMBYX BATRYTICATUS (Bombyx Batryticatus, Jiangcan, JC), NOTOPTERYGII RHIZOMA ET RADIX (Rhizoma et Radix Notopterygii, Qianghuo, QH), CINNABARIS (Cinnabaris, Zhusha, ZS), ARISAEMATIS RHIZOMA (Rhizoma Arisaematis, Tiannanxing, TNX), GINSENG RADIX ET RHIZOMA (Radix Ginseng, Renshen, RS), ACONITI LATERALIS RADIX PRAEPARATA (Radix Aconiti Lateralis Preparata, Fuzi, FZ), EPHEDRAE HERBA (Herba Ephedra, Mahuang, MH), CINNAMOMI CORTEX (Cortex Cinnamomi, Rougui, RG), ANGELICAE PUBESCENTIS RADIX (Radix Angelicae Pubescentis, Duhuo, DH), PINELLIAE RHIZOMA (Rhizoma Pinelliae, Banxia, BX), BOVIS CALCULUS (Calculus Bovis, Niuhuang, NH), ACHYRANTHIS BIDENTATAE RADIX (Radix Achyranthis Bidentatae, Niuxi, NX), ASARI RADIX ET RHIZOMA (Herba Asari, Xixin, XX), AUCKLANDIAE RADIX (Radix Aucklandiae, Muxiang, MX), REALGAR (Realgar, Xionghuang, XH), and ZAOCYS (Zaocys, Wushaoshe, WSS).

**Table 5:** Statistics of the clinician's information from the Delphi questionnaires.

Information on clinicians	Expert selection criteria	Number of qualified clinicians in the first round of surveys (%)	Number of qualified clinicians in the second round of surveys (%)
Working years	More than 15 years	8 (44)	8 (40)
Professional ranks and titles	Deputy Chief Physician and above	18 (100)	18 (90)
Educational background	Master degree or above	17 (94)	19 (95)
Working department	Department of Encephalopathy or Neurology of TCM	18 (100)	20 (100)
Professional requirements	Internal medicine of TCM	18 (100)	20 (100)
Location area	Province with high incidence of stroke	18 (100)	20 (100)
Hospital level	Grade III Hospital of TCM	8 (44)	8 (40)

**Table 6:** Distribution of expert opinions on the clinical application of herbal pairs containing TM.

Herbal pairs	Rounds	Maximum values	Minimum values	Median	Mean	Standard deviation (SD)	Full score ratio (%)
TM ⇒ GT	1	5	4	4	4.33	0.49	100.0
TM ⇒ MJZ	2	5	1	4.5	4.33	0.97	100.0
TM ⇒ JMZ	2	5	0	4	4.11	1.18	100.0
TM ⇒ BS	1	5	3	4	4.22	0.55	95.0
TM ⇒ JH	2	5	4	5	4.72	0.46	94.4
TM ⇒ SJM	2	5	3	5	4.56	0.62	94.4
TM ⇒ DZ	1	5	3	4	3.94	0.42	89.0
TM ⇒ CX	1	5	3	4	4.11	0.58	89.0
TM ⇒ SCP	2	5	3	4	4.39	0.61	88.9
TM ⇒ DL	1	5	3	4	3.89	0.47	83.0
TM ⇒ NX	1	5	1	4	3.78	0.94	83.0
TM ⇒ BX	1	5	1	4	3.67	0.97	83.0
TM ⇒ JC	1	4	3	4	3.78	0.43	78.0
TM ⇒ QX	1	5	3	4	3.89	0.58	78.0
TM ⇒ CS	1	5	1	4	3.78	0.88	78.0
TM ⇒ SM	2	4	0	3	2.39	1.42	72.2
TM ⇒ CP	2	5	0	4	3.50	1.58	72.2
TM ⇒ ML	2	5	0	4	3.39	1.69	72.2
TM ⇒ DNX	1	4	1	4	3.61	0.78	72.0
TM ⇒ GG	2	5	4	5	4.61	0.50	66.7
TM ⇒ CWJ	2	5	0	4	3.56	1.62	66.7
TM ⇒ QH	1	4	3	4	3.56	0.51	56.0
TM ⇒ DH	1	5	1	4	3.33	0.97	50.0
TM ⇒ SQ	2	5	0	4	3.39	1.54	50.0
TM ⇒ XX	1	4	1	3	3.22	0.81	39.0
TM ⇒ BFZ	1	4	1	3	3.17	0.86	39.0
TM ⇒ FF	1	4	1	3	3.17	0.86	39.0
TM ⇒ TNX	1	4	1	3	2.83	0.99	33.0
TM ⇒ MX	1	4	1	3	2.89	0.90	28.0
TM ⇒ SR	2	5	0	3.5	2.94	1.51	27.8
TM ⇒ FZ	1	4	1	3	2.83	0.86	22.0
TM ⇒ MH	1	4	1	3	2.61	0.85	17.0
TM ⇒ RS	1	4	1	3	2.61	0.85	17.0
TM ⇒ NH	1	4	1	3	2.61	0.78	11.0
TM ⇒ RG	1	4	1	3	2.67	0.69	6.0
TM ⇒ SX	1	5	1	3	2.56	0.92	6.0
TM ⇒ XH	1	3	1	2	2.17	0.51	0.0
TM ⇒ ZS	1	3	1	2	2.17	0.51	0.0
TM ⇒ BP	1	3	1	2	2.39	0.61	0.0

Notes: only high-frequency herbal pairs containing TM are shown. The names of Chinese herbal medicines are expressed as medical names, Latin names, Pinyin names, and abbreviations. UNCARIAE RAMULUS CUM UNCIS (Ramulus Uncariae Cum Uncis, Gouteng, GT), VITICIS FRUCTUS (Fructus Viticis, Manjingzi, MJZ), CASSIAE SEMEN (Semen Cassiae, Juemingzi, JMZ), PAEONIAE RADIX ALBA (Radix Paeoniae Alba, Baishao, BS), CHRYSANTHEMI FLOS (Flos Chrysanthemi, Juhua, JH), HALIOTIDIS CONCHA (Concha Haliotidis, Shijueming, SJM), EUCOMMIAE CORTEX (Cortex Eucommiae, Duzhong, DZ), CHUANXIONG RHIZOMA (Rhizoma Ligustici Chuanxiong, Chuanxiong, CX), ACORI TATARINOWII RHIZOMA (Rhizoma Acori Tatarinowii, Shichangpu, SCP), PHERETIMA (Lumbricus, Dilong, DL), PAEONIAE RADIX RUBRA (Radix Paeoniae Rubra, Chishao, CS), SAPPAN LIGNUM (Lignum Sappan, Sumu, SM), CITRI RETICULATAE PERICARPIUM (Pericarpium Citri Reticulatae, Chenpi, CP), OSTREAE CONCHA (Concha Ostreae, Muli, ML), ARISAEMA CUM BILE (Rhizoma Arisaematis Cum Bile, Dannanxing, DNX), PUERARIAE LOBATAE RADIX (Radix Puerariae, Gegen, GG), ACANTHOPANACIS SENTICOSI RADIX ET RHIZOMA SEU CAULIS (Radix et Caulis Acanthopanacis Senticosi, Ciwujia, CWJ), NOTOGINSENG RADIX ET RHIZOMA (Radix Notoginseng, Sanqi, SQ), AMOMI FRUCTUS (Fructus Amomi Villosi, Sharen, SR), and BORNEOLUM SYNTHETICUM (Borneolum Syntheticum, Bingpian, BP).

**Table 7:** Coefficient of variation in the two rounds of the expert questionnaire.

Herbal pairs in the first round	CV (%)
TM ⇒ DZ	10.55
TM ⇒ GT	11.2
TM ⇒ JC	11.32
TM ⇒ DL	12.12
TM ⇒ BS	12.99
TM ⇒ CX	14.18
TM ⇒ QH	14.38
TM ⇒ QX	14.99
TM ⇒ DNX	21.53
TM ⇒ CS	23.25
TM ⇒ XH	23.74
TM ⇒ ZS	23.74
TM ⇒ NX	24.96
TM ⇒ XX	25.09
TM ⇒ BP	25.44
TM ⇒ RG	25.72
TM ⇒ BX	26.45
TM ⇒ BFZ	27.08
TM ⇒ FF	27.08
TM ⇒ DH	29.11
TM ⇒ NH	29.78
TM ⇒ FZ	30.27
TM ⇒ MX	31.16
TM ⇒ MH	32.55
TM ⇒ RS	32.55
TM ⇒ TNX	34.78
TM ⇒ SX	36.06
TM ⇒ JH	9.76
TM ⇒ GG	10.88
TM ⇒ JMZ	13.93
TM ⇒ SCP	13.85
TM ⇒ SJM	13.51
TM ⇒ MJZ	22.39
TM ⇒ CP	25.34
TM ⇒ CWJ	25.82
TM ⇒ SQ	25.73
TM ⇒ ML	32.07
TM ⇒ SR	34.36
TM ⇒ SM	44.48

**Table 8:** Weightiness analysis of herbal pairs containing TM.

Herbal pairs	Dimensionless value of the data mining results (*y*_*iA*_: 0∼1)	Dimensionless value of the statistical results of expert survey (*y*_*iB*_: 0∼1)	Weight value (*W*_*I*_: 0∼1)	Source
TM ⇒ QX	0.868	0.674	0.771	Data mining
TM ⇒ FF	1.000	0.391	0.696	Data mining
TM ⇒ JC	0.685	0.630	0.658	Data mining
TM ⇒ CX	0.501	0.761	0.631	Data mining
TM ⇒ QH	0.674	0.544	0.609	Data mining
TM ⇒ BFZ	0.699	0.391	0.545	Data mining
TM ⇒ JH	0.019	1.000	0.510	Expert provision
TM ⇒ GG	0.021	0.957	0.489	Expert provision
TM ⇒ MJZ	0.105	0.848	0.477	Expert provision
TM ⇒ SCP	0.081	0.870	0.476	Expert provision
TM ⇒ BX	0.354	0.587	0.471	Data mining
TM ⇒ SJM	0.000	0.935	0.468	Expert provision
TM ⇒ NX	0.289	0.630	0.460	Data mining
TM ⇒ GT	0.052	0.848	0.450	Data mining
TM ⇒ BS	0.065	0.804	0.435	Data mining
TM ⇒ TNX	0.603	0.261	0.432	Data mining
TM ⇒ SX	0.710	0.152	0.431	Data mining
TM ⇒ DH	0.356	0.456	0.406	Data mining
TM ⇒ JMZ	0.000	0.761	0.381	Expert provision
TM ⇒ DZ	0.066	0.695	0.381	Data mining
TM ⇒ DL	0.055	0.674	0.365	Data mining
TM ⇒ RS	0.536	0.174	0.355	Data mining
TM ⇒ XX	0.286	0.413	0.350	Data mining
TM ⇒ DNX	0.128	0.565	0.347	Data mining
TM ⇒ CS	0.059	0.630	0.345	Data mining
TM ⇒ FZ	0.416	0.261	0.339	Data mining
TM ⇒ CP	0.112	0.522	0.317	Expert provision
TM ⇒ ZS	0.614	0.000	0.307	Data mining
TM ⇒ RG	0.390	0.196	0.293	Data mining
TM ⇒ MH	0.407	0.174	0.291	Data mining
TM ⇒ MX	0.282	0.283	0.283	Data mining
TM ⇒ CWJ	0.000	0.543	0.272	Expert provision
TM ⇒ NH	0.350	0.174	0.262	Data mining
TM ⇒ ML	0.000	0.478	0.239	Expert provision
TM ⇒ SQ	0.000	0.478	0.239	Expert provision
TM ⇒ SR	0.000	0.304	0.152	Expert provision
TM ⇒ XH	0.264	0.000	0.132	Data mining
TM ⇒ BP	0.090	0.087	0.089	Data mining
TM ⇒ SM	0.000	0.087	0.044	Expert provision

**Table 9:** Information on core targets relevant to TM-CX.

Target name	Protein name	Degree	Average shortest path length	Betweenness centrality	Closeness centrality	Clustering coefficient	MCODE score
PTGS2	Prostaglandin G/H synthase 2	7	1.36	0.22	0.73	0.38	1.57
NOS2	Nitric oxide synthase, inducible	3	2.00	0.00	0.50	1.00	1.80
NOS1	Nitric oxide synthase, brain	3	2.00	0.00	0.50	1.00	1.80
APP	Amyloid-beta precursor protein	8	1.27	0.43	0.79	0.25	1.80
F2	Prothrombin	4	1.64	0.18	0.61	0.50	1.80
ACE	Angiotensin-converting enzyme	6	1.45	0.16	0.69	0.47	1.57

**Table 10:** Information on the core targets relevant to TM-JH.

Target name	Protein name	Degree	Average shortest path length	Betweenness centrality	Closeness centrality	Clustering coefficient	MCODE score
APP	Amyloid-beta precursor protein	6	1.25	0.24	0.80	0.47	1.80
ACE	Angiotensin-converting enzyme	6	1.25	0.15	0.80	0.60	1.61
PTGS2	Prostaglandin G/H synthase 2	6	1.25	0.17	0.80	0.53	1.71
MMP9	Matrix metalloproteinase-9	5	1.38	0.06	0.73	0.70	1.71
LDLR	Low-density lipoprotein receptor	4	1.50	0.06	0.67	0.67	1.80
NOS2	Nitric oxide synthase, inducible	4	1.63	0.01	0.62	0.83	1.80
NOS1	Nitric oxide synthase, brain	3	1.75	0.00	0.57	1.00	1.80

## Data Availability

The data used to support the findings of this study are available from the first author upon request.
